# Dry EEG Electrodes

**DOI:** 10.3390/s140712847

**Published:** 2014-07-18

**Authors:** M. A. Lopez-Gordo, D. Sanchez-Morillo, F. Pelayo Valle

**Affiliations:** 1 ISAEAR Department, University of Cadiz, Cadiz 11002, Spain; E-Mail: daniel.morillo@uca.es; 2 Nicolo Association, Av. Cristobal Colon, Churriana de la Vega 18194, Spain; 3 TSTC Department, University of Granada, Granada 18012, Spain; 4 ATC Department & CITIC-UGR, University of Granada, Granada 18012, Spain; E-Mail: fpelayo@ugr.es

**Keywords:** EEG, dry electrodes, benchmarking, brain-computer interface

## Abstract

Electroencephalography (EEG) emerged in the second decade of the 20th century as a technique for recording the neurophysiological response. Since then, there has been little variation in the physical principles that sustain the signal acquisition probes, otherwise called electrodes. Currently, new advances in technology have brought new unexpected fields of applications apart from the clinical, for which new aspects such as usability and gel-free operation are first order priorities. Thanks to new advances in materials and integrated electronic systems technologies, a new generation of dry electrodes has been developed to fulfill the need. In this manuscript, we review current approaches to develop dry EEG electrodes for clinical and other applications, including information about measurement methods and evaluation reports. We conclude that, although a broad and non-homogeneous diversity of approaches has been evaluated without a consensus in procedures and methodology, their performances are not far from those obtained with wet electrodes, which are considered the gold standard, thus enabling the former to be a useful tool in a variety of novel applications.

## 1. Introduction

The German psychiatrist Hans Berger in 1924 recorded for the first time electrical activity in humans by means of electrodes attached to the scalp and a galvanometer. In 1929, and despite the rudimentary tools and devices at the time, he was able to describe some low-frequency oscillations that he called Alpha waves [1]. After these milestones, the principles and basic procedures of electroencephalography (EEG) have barely changed.

In the EEG acquisition, the preparation time is a laborious process that begins with the localization of sites for the electrical montage. Then, and in order to decrease the skin impedance to acceptable values below 20 KΩ, these locations are rubbed with an abrasive paste that removes part of the outer skin, otherwise called stratum corneum (SC). The SC is the mayor contributor to the skin impedance, with a frequency response that ranges between 200 KΩ and 200 Ω at 1 Hz and 1 MHz, respectively, for a square centimeter [2], although some authors state it to be up to 1 MΩ [3]. Then, popular Ag/AgCl electrodes are impregnated with an electrolyte gel that facilitates the transduction of the ionic currents, which freely move through brain tissues and the cerebrospinal fluid, into electric currents. Furthermore, the electrode-skin impedance must be measured to guarantee a low value.

These are mainly hands-on tasks that require staff with expertise in EEG. Another remarkable inconvenience is the annoyance caused to the subject under test. For instance, the abrasive paste and the electrolyte gel, despite being minimally invasive and barely harmful, are sticky products that make the hair and scalp dirty. Also, the time needed to reduce the impedance to an acceptable value, typically 5–20 KΩ [4], can take a long time. The use of a massive electrolyte to speed up the impedance reduction could cause electrical bridges between electrodes, especially with dense arrays, thus being counterproductive. However, these are not the only problems. Once an acceptable electrode impedance has been achieved, a countdown begins until the gel dries, thus causing the transductive properties to disappear. For instance in [5], the impedance of wet electrodes deteriorated from 5 to 15 KΩ within 5 hours after gel application. For these reasons, wet electrodes are not suitable for long-term measures [6].

In recent decades, there have been several approaches to develop dry electrodes based on microelectro-mechanical systems (MEMS), non-contact, capacitive, *etc.* These approaches, when combined with the new generation of on-site low energy instrumentation amplifiers, enable the development of portable, active and dry electrodes in a back-to-back design. Therefore, dry and active electrodes seem to be the solution to the disadvantages of wet EEG electrodes. However, these new technologies must be conveniently evaluated and validated before use.

Wet electrodes are considered the gold standard and new dry approaches must be compared with them before claiming that they are suitable. The first difficulty is to agree on the evaluation procedure. Different dry electrode approaches are conceptually different and, in the literature, reports of performance have been carried out without homogenous methodologies, so that their results cannot easily be compared. These studies and reports show evidences that additional work is needed before dry electrodes become an alternative to standard wet electrodes for the recording of EEG signals in clinical and other applications with long-term exposures.

## 2. Basic EEG Acquisition Principles

The electroencephalogram (EEG) is a record of the oscillations of brain electric potentials acquired from electrodes on the human scalp [4]. Electric potentials are the direct consequence of the existence of electric dipoles created by the postsynaptic potentials generated at apical dendrites of pyramidal cells in the cortex. The poles of the electric dipole can be seen as the source and sink of ionic currents created by the excess and defect of cations at soma and apical dendrites, respectively. These ions can freely move through the cerebrospinal fluid and brain tissues, thus causing ionic currents as providing the most accurate evidence of the existence of electrical potentials.

**Figure 1.**
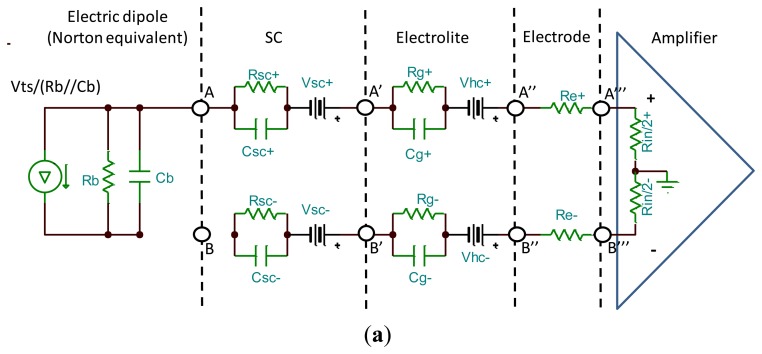

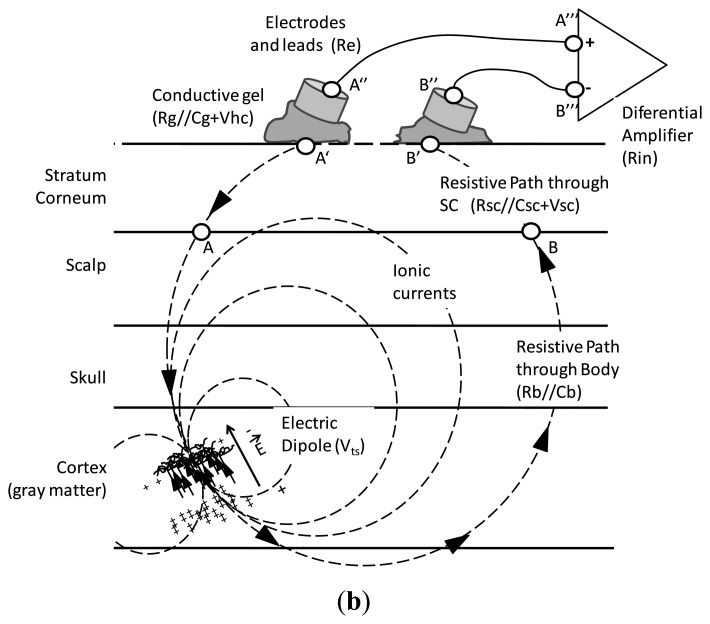
(**a**) Simple model of the electrical circuit originated by the electric dipole in a differential montage; (**b**) Schema of electric dipole, ionic currents and differential measure.

These potentials can be measured by attaching a voltage meter to any two points of an ionic current line. In a non-invasive approach, two locations on the scalp are chosen and a conductive gel is applied between the skin and the electrode for an efficient charge transduction. Figure 1a shows a simple model of the electrical circuit originated by the electric dipole of Figure 1b. The electrode-skin impedance is electrically characterized by the half-cell voltage source in series with the gel impedance (*Vhc* + *Rg*‖*Cg*) plus the SC impedance (*Vsc* + *Rsc*‖*Csc*). In this model, the SC offers the largest impedance while impedance due to conductive gel, electrodes and copper leads are much lower.

Typical EEG electrodes have two major mechanical and electrical restrictions, namely the size and impedance. The electric dipole caused by just one pyramidal cell cannot be measured with electrodes attached to the scalp. However, when a large number of dipole units, approximately 60 million [4], synchronously discharges their action potentials, it gives rise to potentials in the scale of microvolts, large enough to be measured with non-invasive methods. In summary, the EEG is the macroscopic measure of the synchronous activity of a large population of neurons. A theoretical estimate of the area required to cover this population is approximately 6 cm^2^. Current implementations are not far from this estimate. A typical diameter of a EEG electrode is 10 mm (1.6 cm^2^) and commercial products such as Quick-Cap use an effective size of 7 mm [7]. In summary, innovative-dry approaches suffer from a severe restriction for miniaturization.

The first obvious disadvantage when neither conductive gel nor abrasive paste are used is larger electrode impedance. Typical values of the electrode-skin impedance range between 150 to 200 KΩ and 5 to 10 KΩ before and after gel applications, respectively. There are reasons why this impedance must be kept as low as possible. The first one is to reduce the negative effect of the voltage splitter at electrical nodes *A*‴ and *B*‴ (see Figure 1a). The second is to reduce undesired effects of mismatch impedances in differential EEG measures.

Differential measurements are a popular way to record EEG because they prevent artifacts caused by electromagnetic interference (EMI), mainly by power lines. EEG front-end amplifiers normally present very high common-mode rejection ratio (CMRR) with typical values above 100 dB. The CMRR consists of the ability of the amplifier to attenuate identical signals that simultaneously appear at the inputs of the amplifier. EMI is mainly coupled to the skin and the electrode leads. Therefore, and providing that electrode leads offer exactly the same impedance to both inputs, it is presented at the amplifier inputs as a component of the common mode. In this case, it is almost completely removed by the front-end differential amplifier.

## 3. Dry Electrodes

In the literature, dry electrode approaches can be classified as spiky, capacitive, non-contact or other heterogeneous approaches.

### 3.1. Spiky Contact

In this approach, the electrode surface consists of an array of spikes that either directly come into contact with the scalp or pierce the SC for better electrical attachment and mechanical fixation to the scalp. Spikes have been developed in the scale of nanometers, micrometers (MEMS) and millimeters.

#### 3.1.1. Nano, Micro and Millineedles

**Figure 2.**
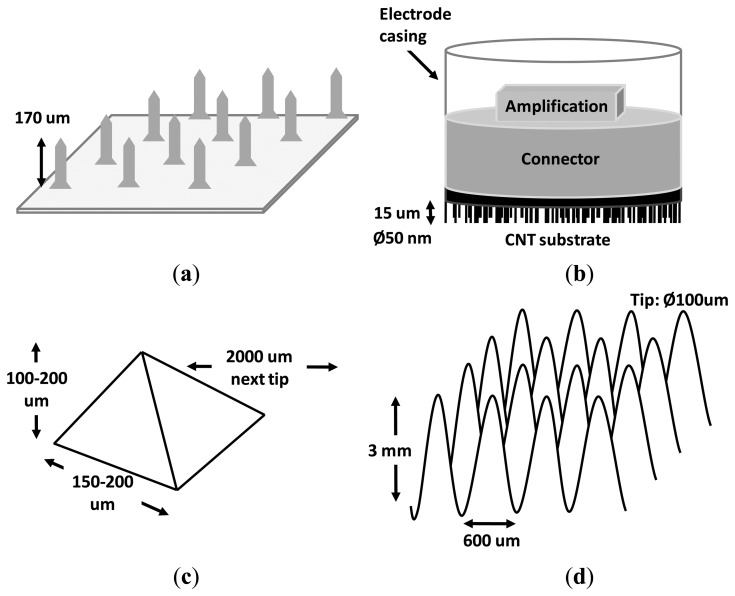
(**a**) Array of silicon microneedles. Adapted from [8]; (**b**) Multiwalled carbon nanotube arrays. Adapted from [11]; (**c**) Details of a microtip. Adapted from [12]; (**d**) 3D printed dry millielectrode. Adapted from [16].

In [8], an array of silicon microneedles were tested for an EEG recording during anaesthesia monitoring (see Figure 2a). Electrode configurations with different aspects and characteristics were analyzed, revealing that the most relevant factors that affected the impedance were the electrode size and the coating material. For example, Ag/AgCl-coated electrodes showed significantly lower impedance than Ag-coated ones (*p* < 0.01). As in the case of disc-type Ag/AgCl electrodes, the impedance increased with the decrease of the electrode size. Adequate impedance for EEG measurements was obtained with an electrode array of just 3 × 3 mm^2^. The length of the spikes, which is a key factor in the design because it determines the fragility of the electrode, was also analyzed finding no significant difference between 200 and 170 μm (*p* > 0.3). With the best configuration (Ag/AgCl-coated, 3 × 3 mm^2^), the minimum impedances were 0.65 KΩ and 16 KΩ at 1 KHz and 0.6 Hz, respectively. Regarding the quality of the recorded signal, EEG recordings with commercial Zipprep electrodes showed similar quality by means of visual inspection. The study concluded that the microneedle array was convenient for the patient and also suitable for real-time, long-term EEG recordings. This approach, however, has a disadvantage, namely, the strength of the needles. Although in the study a low rate of broken spikes was reported (0.3%), when spikes are broken they cause an impedance mismatch in differential measures as well as infections. The latter would justify the sterilization process being carried out three hours previous to the beginning of the experiment. From the usability point of view, this process is obviously less convenient than wet electrodes and not likely to be used in non-clinical applications, such as brain–computer interfaces (BCIs) [9,10]. Another relevant aspect to be considered is that electrodes were located on the forehead for sake of the anaesthesia monitoring (the fixation mechanism was not reported). Therefore, limitations due to the presence of hair were not evaluated.

Six years later, multiwalled carbon nanotube arrays (see Figure 2b) were designed to penetrate the SC, resulting in a comfortable and pain-free interface due to the minuscule size of the spikes [11]. The use of this smaller technology (nanotube diameter 50 nm, length 15 μm) resulted in a lower infection risk as compared to micro approaches. The performance and quality of the recorded signal was visually compared with a commercial wet EEG system, showing similar results in both the frequency and time domains. Although it was stated that the nanotubes could be coated to improve the transduction from ionic to electronic currents, the electrode impedance was not reported. This missing information is critical to evaluate the relative effect of both the input impedance of the front-end amplifier and the mismatch impedance. An aspect in common with [8] is that the position of the dry electrode during the evaluation protocol was in the forehead (Fp2 position in the International 10–20 system) with the reference at the nose. Again neither the disturbance due to the presence of hair was evaluated nor was the fixation mechanism specified (from the context it is assumed that headbands or a helmet was used).

Coming back to the micro range in 2010, a new dry electrode composed of an array of 4 × 4 microtips on silicon substrate coated with iridium oxide was presented [12]. The most remarkable novelty of this dry electrode was its capability for both recording and electro-tactile stimulation. The use of iridium oxide is a promising material for stimulation that enables high charge delivery capacity and low constant impedance over the entire frequency range [13]. The electrode was not electrically characterized in an *in vivo* experiment, but in a back-to-back montage (*i.e.*, electrode-get-electrode montage, see Figure 6b) that showed a conductivity of 5–14 mS/cm and linear phase from approximately 4 Hz onwards. Signals recorded with this electrode were visually compared with wet electrodes in both the frequency and time domain, giving rise to similar results. Due to the fragility of the tip and the necessary pressure to puncture the scalp, some of the tips are expected to break during skin penetration, especially those with aspect ratios higher than the proposed electrode, namely 150–200 μm base, 100–200 μm height, 54.7° angle (see Figure 2c). As with the two previous studies, measures were performed on places without hair. Actually, in this study, no EEG recording was performed, but EOG, for which electrodes were located at the canthi of the eyes. The amplitudes of the EOG signals recorded in the study were about two orders of magnitude above typical EEG amplitudes (e.g., hundreds of μV *vs.* units of μV). In summary, in this study neither the influence of hair nor the suitability to record EEG signals was evaluated.

Other studies, such as in [14], reported that MEMS dry electrodes had several advantages in comparison with wet ones, such as the electrode–skin interface impedance, signal intensity and size of the electrode. Each electrode consisted of an array of 20 × 20 micro probes coated with Titanium/Platinum (each probe 250 μm height, 35 μm diameter, 200 μm of effective penetration). The length of the probes was not enough for recording on hairy sites. Therefore, only Fp1 and Fp2 positions were tested. In addition, they also developed a driver's drowsiness estimation system to demonstrate the potential applications of the MEMS electrodes in operational applications.

The principle of the nano and microneedle approach is the ability to by-pass the SC, gaining access to inner layers of the skin with lower impedance. However, these microscopic structures frequently split, giving rise to infections and impedance mismatch between electrodes. In 2012, Forvi E. *et al.* [3] carried out a technological assessment of microneedle-based dry electrodes that do not break when piercing the SC. In this study, they produced a dry EEG electrode of 8 × 8 pyramidal microneedles, hosted in an area as little as 10 mm^2^, with sharper microtips that facilitated SC piercing, thus avoiding tips breakings. Once the electrode pierced the SC, it achieved an impedance of just 13 KΩ without any tips failing by the end of the experiment. They reported ECG, EMG and EEG validation experiments. In the EEG experiment, the methodological section states that electrodes were located at position Fp1, Fp2, T3 and T4 of the 10–20 system (non-hairy and hairy sites, respectively) with ground and reference at Fpz. However, in the results sections, only signals recorded using Fp2 (site without hair) were analyzed. Therefore, and as in the studies previously reviewed, limitations due to the presence of hair were not evaluated.

The approaches previously described in this section, based on nano and microneedles, are alternatives to wet electrodes. They present low electrode impedance, high mechanical stability with fewer artifacts due to motion, capability for long-term measures and a quick set up. However, these approaches are not easy or cheap to produce. Moreover, the electrode fixation was not always specified and none of them was evaluated in areas covered with hair. A justification for the latter resides in the fact that the size of the tips has to maintain the balance between invasiveness, reliability against breaks and the capability to complete SC piercing. The SC has a thickness of 10–40 μm, whereas a human hair is on average 50–100 μm [15]. That means that typical tips of 100 μm should be enlarged by at least 200–300 μm extra to enable complete SC piercing in areas covered by abundant hair. As a consequence, an electrode of this length would risk being both invasive and fragile.

In order to avoid the problems of the nano and micrometer scale, a non-invasive variant of the microscopic spikes was developed in the millimeter scale. In [16], a 3D printer with micrometric resolution was employed to make a dry electrode composed of 180 conical needles treated with titanium and gold to lower the impedance and prevent oxidation (see Figure 2d). This approach permitted a fast and low-cost production with high precision, thus being immediately ready for small scale home production. This approach is not invasive at all, and can be reused indefinitely seemingly without maintenance. The needles are much bigger than the nano or micro needles mentioned previously. In this study, each needle was 3 mm long, 600 μm base diameter and about 100 μm tip diameter. Furthermore, the distance between needles, namely 250 μm, permitted the operation on hairy sites without compromising the fragility or invasiveness of needles. The millineedle electrode was visually compared in the frequency and time domain with gel-based electrodes showing similar results. One advantage of this approach is that, due to their size, EEG measures can be performed on areas with hair (e.g., the occipital area). One logical disadvantage of the millimeter scale approach is the likely motion artifacts due to inadequate contact/attachment with skin since millineedles do not pierce the SC. It could be solved by attaching the electrodes to a belt and fastening it to the user's head. Nevertheless, this method could be unpleasant and even painful for the patient. Instead, it is believed that an *ad hoc* cap with integrated electrodes would ensure a good contact with the skin. Another disadvantage is a higher electrode impedance, which approximately reaches 50 KΩ for the lower part of the EEG spectrum (DC-20 Hz).

As a summary, dry electrodes based on needles and their respective variants are approaches that conveniently avoid the need for conductive gel and fixation paste. Cheap and minimally invasive electrodes can be easily obtained by means of a 3D printer, with low risk of infection and work on hairy sites in the millimeter scale. However, this solution is not optimal from the impedance point of view, for which subscales could be tried.

#### 3.1.2. Tips

**Figure 3.**
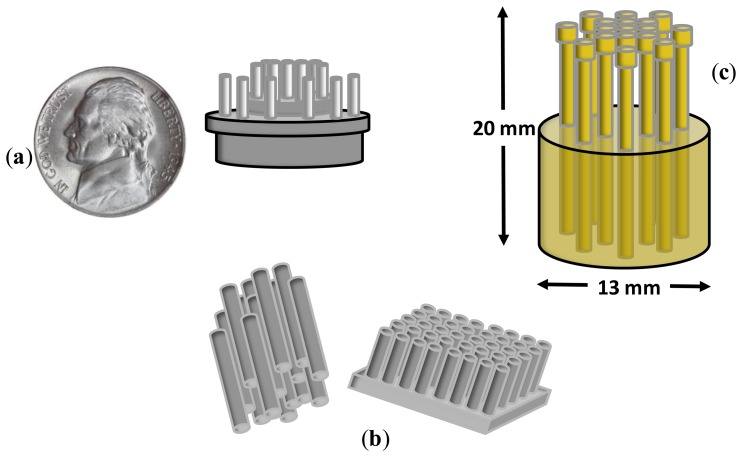
(**a**) Ambulatory Wireless EEG System Adapted from [17]; (**b**) Polymer bristles. Adapted from [18]; (**c**) Spring contact probes. Adapted from [19].

In the centimeter scale, Matthews R. *et al.* [17] proposed an electrode of the size of a US 5c coin based on a set of tips (*i.e.*, ‘fingers’) is proposed which would be big enough to make electrical contact with the scalp through hair. The contact impedance between the scalp and each finger was as high as 10 MΩ. In order to reduce this undesired effect, the electrode was directly connected, back-to-back to an amplifier of ultra-high input impedance. The tips were allocated in two concentric rings (see Figure 3a) for a better rejection of common mode. The signal quality was visually compared with wet electrodes showing similar recordings with closed eyes. Also, real time classification of workload and engagement was performed as a measure of the ability to extract useful information from EEG recordings, obtaining accuracies between 73% and 89% in binary classification. An EEG harness fixed the electrodes in hairy positions. It can be argued that, due to the high electrode impedance, on-site amplifier with ultra-high impedance is required. The other aspect to keep in mind is that the comparison with wet electrodes was performed on EEG envelopes of 50 μV peak-to-peak. Thus, the performance of small potential recordings was not shown.

Three years after in 2011, the authors of [18] developed a flexible, low-cost electrode made of polymer silver-coated bristles approximately the size of a toothbrush (see Figure 3b). The electrodes were evaluated with typical BCI paradigms, namely motor imagery, odd-ball paradigm, as well as with EEG components, namely auditory evoked potentials and P300. The results confirmed the ability of these electrodes to record EEG signals with enough quality to be used in a wide variety of BCI applications and EEG analysis. One of the disadvantages of this electrode is the rapid deterioration of the electrode impedance with use. An initial impedance of 80 KΩ was reported that deteriorated to 150–200 KΩ after 10 months of use. At this point, all electrodes must be recoated in order to avoid impedance mismatch. Another disadvantage is the dependence of the electrode impedance with the pressure on the scalp. Some participants reported prickling and uncomfortable sensations.

Although the bristle-sensor improved the risk of infection, degree of invasiveness and surface contact, it was too invasive and uncomfortable for long-term measures. In [19], the authors presented a dry EEG sensor for operation in the presence of hair. This sensor was designed to contact the scalp surface with 17 spring contact probes that kept high geometric conformity between the sensor and the irregular scalp surface, thus maintaining low electrode impedance (see Figure 3c). Additionally, the flexible substrate in which the spring probes were inserted permitted the attachment of the sensor to the scalp without pain when force was applied. It was compared with wet electrodes, achieving similar results in terms of signal quality recordings and electrode impedance with a better temporal derive, thus enabling long-term EEG records.

### 3.2. Capacitive/Non-Contact Electrodes

In previous sections, we pointed out that hair can be an inconvenience with nano and microelectrodes as it can cause loss of contact with the scalp. Some researchers coped with this difficulty by avoiding physical contact with the scalp, but at the cost of an extraordinary increase of electrode impedance.

In [20], the authors recorded EEG electric potential with probes at a distance of 3 mm from the scalp above positions P3-O1 of the International 10–20 System. The probes fed an amplifier whose ultra-high impedance avoided additional degradation of the measured EEG signal. This study has some shortcomings. The most important one is that it cannot be reproduced, since no details were provided about the electrodes design or the acquisition system. Furthermore, there was no comparison with wet electrodes, but a demonstration that the electrode was able to record modulations of the Alpha rhythm in a closed–open eyes paradigm.

In [21], the authors presented a dry and non-contact EEG/ECG sensor that combined amplification, filtering and analog-to-digital conversion within an enclosure of the size of a quarter dollar. The measured input-referred noise, in the range 1–100 Hz varied from 2 to 17 μVrms at 0.2 mm and 3.2 mm distance, respectively. The same criticism as in [20] can be stated, namely, the EEG was recorded through hair, but the ability of the electrode to discriminate between open and closed conditions from Alpha band was only analyzed without comparison with wet electrodes.

**Figure 4.**
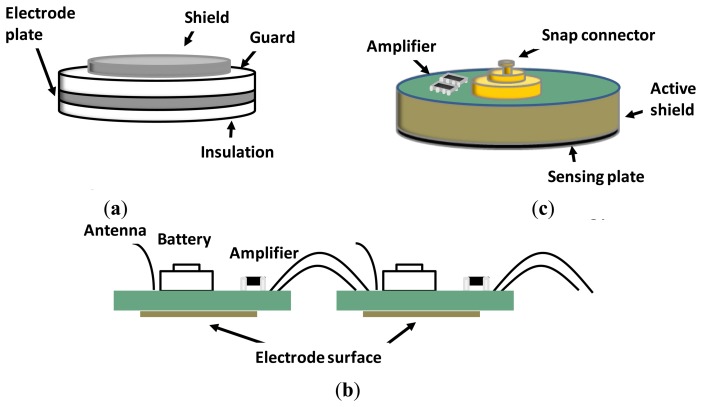
(**a**) Capacitive electrode. Adapted from [22]; (**b**) Non-contact low Power EEE/ECG electrode. Adapted from [23]; (**c**) Dry active electrode made from standard Printed Circuit Board (PCB). Adapted from [24].

The first study of capacitive electrodes related to BCIs [22] presented a helmet of 28 EEG channels that measured steady-state visual evoked potentials (SSVEPs) through hair (see Figure 4a). Although the detection time deteriorated by about a factor of ×3 in comparison with contact electrodes measures, the peak performance, namely ITR = 12.5 bits/min and accuracy = 95%, was far above the minimum accepted by the BCI community, namely 70% [25], to establish an efficient BCI communication session. This approach has the following in common with the non-contact designs previously analyzed in this section: (i) a very high impedance (approximately 10^12^ KΩ), (ii) the need for on-site amplification and (iii) the recorded EEG signal was not compared with that obtained from wet electrodes. Therefore, the scope of application of this electrode cannot be generalized beyond SSVEP-BCI applications with low performance.

In [23], the authors developed a non-contact capacitive biopotential electrode with the capacity for cooperative work in a body area sensor network. The sensor network utilized a conductive plane to establish a common reference, thus eliminating the need for explicit ground. Each electrode was the size of a coin and the network could be fed with a single 3.3 V supply. The integration on the net was performed via a serial daisy chain bus (see Figure 4b). The electrodes were tested at a 0.18, 0.89 and 2.72 mm distance from the scalp, with an approximately linear phase with the frequency and independently of the distance. The gain was distance dependent. With regard to the EEG-signal-quality evaluation, unfortunately, there was neither comparison with wet electrodes nor discrimination between conditions (e.g., open-closed eyes). The study only reported noise spectrum at different distances from the scalp in the band 0.1–150 Hz. In [24], the same author reviewed a simple implementation of a dry active electrode made of conventional PCB. This design has the capability of working as capacitive (see the sensing plane on the bottom of Figure 4c) or as non-contact through insulation fabric (e.g., cotton).

In summary, there are some major drawbacks for the use of capacitive/non-contact EEG electrodes. Firstly, the amplitude levels are so low that they are probably not suitable for recording of spontaneous EEG signals. Secondly, there is the high impedance that these electrodes present, thus causing the need for an on-site amplifier with ultra-high impedance. In this case, and according to the Johnson-Nyquist definition of thermal noise [26,27], the level of electrical noise introduced is proportional to the magnitude of the impedances, thus causing the immediate degradation of the signal quality at the amplifier output. These two drawbacks would justify the use of Alpha and SSVEP for evaluation in all the reviewed studies. These are narrow-band rhythms of inherent high SNR suitable for recordings in the presence of high level of noise. This would justify why capacitive/non-contact electrodes have not been tried either with spontaneous EEG or event-related potentials. The third main inconvenience is motion artifacts caused for the “floating” fixation to the scalp.

### 3.3. Other Heterogeneous Approaches

**Figure 5.**
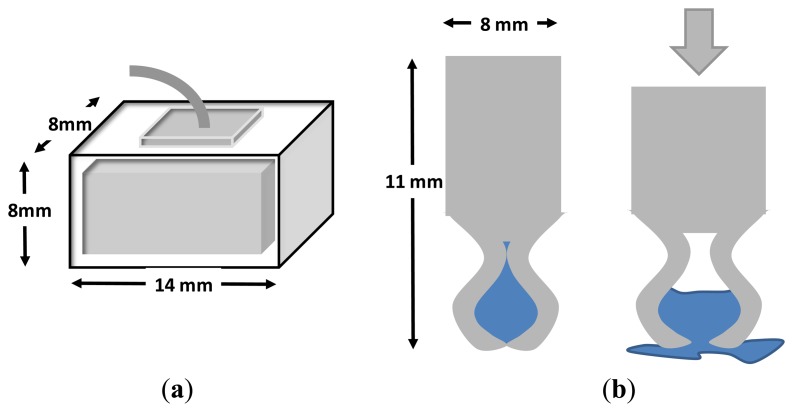
(**a**) Dry foam EEG electrode. Adapted from [5]; (**b**) Quasi-dry electrode Adapted from [28].

Other heterogeneous approaches were tried. In [5], the authors presented a dry electrode with dimensions 14 × 8 × 8 mm, fabricated with electrically conductive polymer foam and covered by a conductive fabric (see Figure 5a). The foam substrate allowed conformity between the electrode and irregular surface of the scalp, thus keeping electrode impedance low, even under motion. It permitted long-term EEG measurements without skin preparation or conduction gel, thus being ideal for daily life applications. The impedance was compared with wet electrodes with and without skin preparation at Forehead (F10) and hairy locations (POz), finding better results for the foam based electrode. Also, the electrode impedance remained approximately the same after 5 h of recording, while wet electrode impedance increases linearly with the time (approximately 2 KΩ/h). Another interesting aspect is the cost of the electrode (around 0.3 euro/unit). Finally, the analysis of motion artifacts was also in favor of the foam electrodes.

In [28], a polymer-based electrode was presented with a reservoir inside the electrode that released a small portion of 30 μL of hydrating agent after an abduction force (see Figure 5b). This electrode was able to monitor EEG signals with similar performance to Ag/AgCl electrodes but using only a fraction of the hydrating solution necessary, thus avoiding dirtying the patient's hair and the risk of conductive bridges between adjacent electrodes. In [29], SRICO Inc. developed dry photonic electrodes, otherwise called “Photrodes” for measuring EEG and ECG signals.

## 4. Benchmarking and Evaluation Procedures

There are inherent problems related to EEG measures that make the evaluation of dry electrodes difficult. Developers of dry-electrodes claim there is no need for conductive and abrasive paste and no degradation of the EEG signal. The latter must be contrasted with the gold standard, namely wet electrodes, by means of a reproducible methodology. In this regards, key aspects such as simulated *versus in vivo* recordings, the nature of the EEG signal (e.g., rhythms, endogenous potentials, event-related potentials, *etc.*), the psycho-physiological paradigm (e.g., odd-ball, steady state response, selective attention, *etc.*) or the electrical montage (e.g., sites, electrical references, devices, *etc.*) must be taken into consideration.

**Figure 6.**
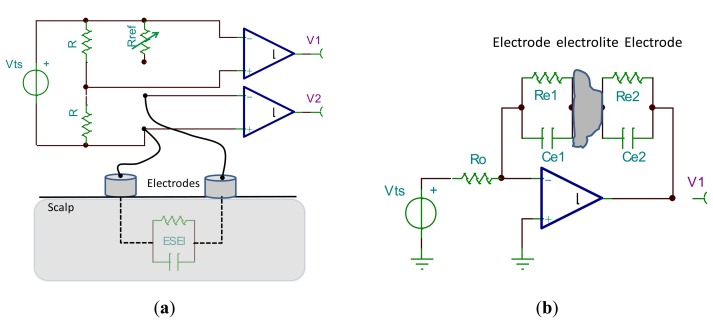

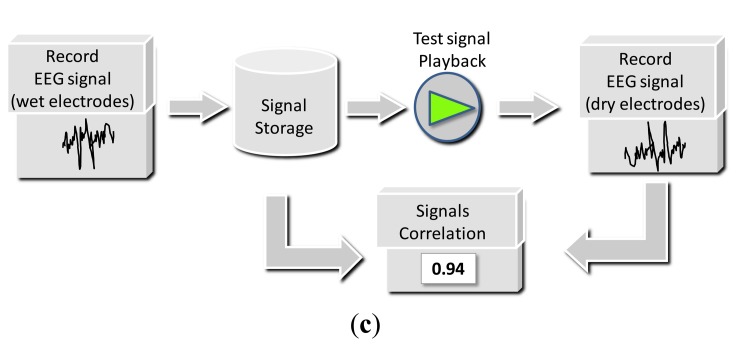
(**a**) Measure of ESEI by means of test signal. Adapted from [30]; (**b**) Impedance-to-voltage converter to measure the EEI. Adapted from [12]. Playback schema.

Figure 6a shows an electrical montage used for electrode characterization [14,30]. In this circuit, a test signal of a known amplitude and phase is generated by the source *Vts.* From the symmetry of the circuit, it can be deduced that the amplitude of output *V_1_* equals that of output *V_2_* when the impedance *Rref* matches the electrode-skin-electrode impedance (ESEI). The phase shift between test signal and V2 will reveal the imaginary part of the ESEI. Typical values for test signal are 60 mV amplitude and frequencies oscillate between 0.5 and 500 Hz. There are other simpler circuits, such as the one proposed in [31]. In this circuit, a known voltage is supplied to all EEG electrodes except one, from which the electrode-skin impedance is estimated. Another circuit proposed in [12] for the measurement of the electrode-electrolyte impedance (EEI) is based on an impedance-to-voltage converter in a configuration with face-to-face electrodes (see Figure 6b). These two montages use a test signal that simulates EEG signals. There are other approaches based on *in vivo* recordings or playback of previously recorded EEG signals.

### 4.1. Simulated, in Vivo and Playback

Unfortunately, there are few studies that perform a complete characterization of the impedance of dry electrodes including frequency response such as in [12], in which the conductivity and phase was estimated by means of impedance spectroscopy in the range 1–1000 Hz. In [32], dry and wet electrodes were compared by applying a test signal of 0.3 V to a pig skin on which both electrodes were placed. Although the performance of the dry electrode was similar to that of the wet, the test signal was generated only at 10 Hz, a reason why the evaluation cannot be considered complete. In [5], signals of 1 V amplitude and frequencies from 0.5 to 10 kHz were employed to determine the impedance by means of impedance spectroscopy. The contrast with wet electrodes showed that the impedance of dry electrodes was approximately the same as the wet ones on non-hairy sites (F10) and better than wet on hairy sites (POz). The study does not specify if the hair was manually removed from the hairy site. A long-term test was performed to evaluate the drying effect on wet electrodes. The results showed that the impedance of the dry electrodes remained constant during 5 hours, while the wet electrode suffered an increment of the impedance at a rate of approximately 2 KΩ per hour. A similar approach and results were reported in [19]. In these two experiments, only the absolute value of the impedance was reported, thus lacking the imaginary part of the frequency response. However, it must be kept in mind that the relevance of the electrode impedance is relative to the input impedance of the front-end amplifier, so the imaginary part of the electrode impedance could be disregarded in the case of much larger amplifier impedance. Impedance is a typical indicator of signal quality when wet electrodes are used; however, when dry electrodes are used the ultimate goal is the recording of signals with the same quality. For this reason, most of the studies do not deal with the electrode impedance question and focus on the quality of the recorded signal.

*In vivo* experimentation is a complicated choice for measures comparison due to the non-reproducible nature of EEG signals. Despite the inherent difficulty of testing with humans, some studies tried to compare the performance of dry electrodes with wet electrodes in a same-place-different-time approach. For instance, in [3] EEG signals were registered on the same locations, namely Fp1, Fp2, T3 and T4 sites, ground and reference at Fpz and right mastoid, respectively, with dry electrodes and afterwards with wet ones. The user was asked to perform eyes blinking and teeth grinding. The same-place-different-time approach is not free from controversy. It can be argued that our brain is in constant change and properties such as plasticity or habituation could drive the participant to different cognitive states and hence to different EEG signals even between consecutive trials of the same experiment.

The same-place-different-time approach *in vivo* experiments has been counteracted with the same-time-different-place approach. Some authors tried an electrical montage in which both dry and wet electrodes are mounted at the same time but at very close locations. In [17], EEG recordings were made by using dry and wet electrode pairs positioned at Cz and Fz sites with less than a 5 mm gap, achieving correlation of dry-wet signals of above 90%. However, there is the possibility that a gap of only 5 mm separation between dry and wet electrodes could easily give rise to electrical bridges due to conductive gel spreading, although the authors claim that care was taken to prevent it. The following studies also followed the same-time-different-place approach to compare dry-wet electrodes [5,11,16,18,19,28]. The same-time-different-place approach is also controversial. On one hand, the use of electrodes at separated locations leads to the measure of different ionic currents and hence different electrical activity. On the other hand, very close located electrodes could lead to electrical bridges caused by the spreading of gel on or even under the scalp. There are studies that report a spread of 1 cm in each direction under the scalp. That gives rise to a 2 cm separation between electrodes [33]. Therefore, a reasonable precaution of 3 cm apart should be kept for sake of isolation. In [34], the authors performed cognitive paradigms such as the oddball and evoked event-related potentials, from which little difference was found in the comparison of dry-wet electrodes. The comparison included accuracy in a single-trial detection of the P300 and, although there was a significant difference in favor of the wet electrodes (77.8% *vs.* 72.1%), the accuracy obtained by the dry EEG system was good enough for efficient communication of a BCI user. The study states that both dry and wet electrodes were recorded simultaneously at the same locations, with only a separation of 1.5 cm. Because the gel spread under the scalp, electrical bridges under the scalp could have occurred, thus explaining the good results with dry electrodes.

Finally, the last evaluation technique is the playback of a previously recorded EEG signal (see Figure 6c). In [5,19], EEG pre-recorded signals by means of standard wet electrodes were stored in a computer. Afterwards, it was played back by means of a function generator and the amplitude divided by approximately a factor of five thousand to accommodate the range of the signal to typical EEG values. The replicated EEG signal was presented to the dry electrodes, and afterwards amplified by the same factor. Finally, both the pre-recorded EEG signal and that recorded with the dry electrodes were statistically compared by means of correlation, obtaining values above 90% and 80% for EEG and EOG signals, respectively.

### 4.2. Evaluation by Means of EEG Rhythms or Evoked Potentials

In the literature, comparison of the recorded signal quality between dry and wet electrodes is based on rhythms and evoked potentials. The adequate recording of a specific EEG rhythm is made with the selection of the psycho-physiological paradigm that better elicits it. Alpha and Beta rhythms are used in literature for the assessment of dry electrodes, although, steady state responses, such as the visual (SVVEP) have also been tried. Some studies used the open–closed eyes task to cause modulations in the energy of Alpha rhythm [3,11,16–18,21,28]. These modulations are large enough to recognize both conditions of the task with dry electrodes, thus facilitating visual contrast between conditions. One of the advantages of the Alpha rhythm is that, due to its large amplitude, it can be recorded all over the scalp (typically in the range of tens of microvolt), thus being a good option in the same-time-different-place approach. In [22], a SSVEP paradigm was proposed for a BCI application. The performance of dry electrodes was evaluated in terms of accuracy in detection of the potential and information transmission rate in bits per minute units. A SSVEP is a train of repetitive evoked responses whose frequency matches that of the visual stimulus. In this paradigm, the electrodes position is important and must preferably be on the visual cortex zone (occipital area). One of the advantages of SSVEP is that, when the SSVEP is optimally evoked, it is a potential of very high signal-to-noise ratio. Under these conditions, the burden of the same-place-different-time approach issue can be overcome by just designing an experiment of short visual stimuli, without significant or cognitive meaning (e.g., non structured stimulus or flash). In this way, cortical adaptation is unlikely to happen while a SSVEP signal is recorded.

Although Alpha rhythm seems to be a convenient solution to test dry electrodes it can be argued that its energy can be easily modulated by cognitive tasks or mental states indicating relaxation or arousal. Then, analysis and comparison of EEG signals recorded in different trials may give rise to misleading conclusions because amplitudes could result from cognitive processes and not the performance of the electrodes. Furthermore, as Alpha rhythm is allocated in a narrow band of the EEG spectrum, the characterization of the electrode, which has a frequency response, is incomplete. SSVEPs have a similar problem with the spectral characterization, which is more prominent for frequencies lower than 30 and typically not beyond 47 Hz [35]. Therefore, electrodes cannot be completely characterized and their performance can be rather different in other spectral bands, such as in the case of slow cortical potentials at 0.01–4 Hz, gamma band from 32 Hz onwards or the multiple auditory steady state responses paradigms about 100 Hz [36]. Despite the incomplete characterization when rhythms or steady state responses are used for the evaluation of dry electrodes, it can be enough for some BCIs applications. Both Alpha and SSVEP can be easily modulated by cognitive tasks, which is why they have been used repeatedly in BCI literature [37,38]. For this reason, the characterization of electrodes by means of EEG rhythms should be linked to the application scope in which these rhythms operate.

The analysis in the frequency domain of EEG signals cannot point out peaks, amplitudes and latencies of evoked responses with accuracy. Some clinical procedures rely on these details for accurate diagnoses, such as the amplitudes and latencies of potentials for visual or auditory impairment assessment. Then, the assessment of dry electrodes in the time domain is pertinent, especially for clinical purpose or for BCIs based on event-related potentials. In the time domain, there are different ways to report similitude between any two signals, such as determination coefficient, correlation coefficient or mean-square error. Only few studies report some statistics, such as correlation [5,6,19,39], or cross-spectrum [18].

## 5. Limitations and Commercial Solutions

In previous sections, we have analyzed the performance and main characteristics of dry electrodes. In this section, we summarize their limitations in comparison with the wet ones as regards the following aspects:

- Mechanical: The size of dry electrodes is not smaller than that of wet, but the spikes that make up the electrode are. The use of smaller spikes that pierce the SC causes the reduction of the electrical impedance at the cost of invasiveness. Table 1 shows some aspects of dry electrodes spike size. The mechanical fixation, which does not differ from the wet ones (e.g., headbands, helmet, *etc.*), needs improvements in terms of comfort and discretion to consider it a wearable solution.
- Evaluation: In some studies, the electrical characteristics of the electrodes, including frequency response, were not reported. Furthermore, comparisons with wet electrodes were not made, and other critical details that would have enabled results to be reproduced were not given in some studies. Instead, in some cases, dry electrodes were validated by visual correlation of specific EEG features (e.g., energy in Alpha band or steady-state evoked potentials) with simple protocols such as open-close eyes or gaze at a flickering stimulus. The latter suggests that the use of some dry electrodes could not be extended beyond certain specific applications (e.g., Alpha-BCIs or SSVEP-BCIs, respectively). Some of the procedures for comparison with wet electrodes are not free from controversy. The same-place-different-time approach compares non-stationary signals recorded at different times. The same-time-different-place approach compares EEG signals mainly generated by different population of neurons (where electrodes are quite separate). The possibility of electrical bridges by gel spreading under the scalp (where electrodes are close together) is always present.
- Usability: Several aspects such as preparation time and comfort, particularly for severely disabled people who have great difficulties in controlling their heads, should be taken into account. Given their current size, dry electrodes are not more comfortable than wet electrodes in this situation. Extra work should be done to develop more comfortable fixation systems other than those already used with wet electrodes. Regarding preparation time, dry electrodes potentially save time for the researcher. However, the time needed to obtain stable signals has not been reported or contrasted with wet ones. For measures that take longer (e.g., video-EEG or sleep sessions), dry electrodes are superior to wet, whose performance deteriorates as the gel dries. Another aspect to consider is the use of active electrodes. Since they convert a high impedance source into a low impedance output, the signal quality is much less skin-impedance dependent. This permits their use without gel. Therefore, the dry-active combination should be considered for a useful reduction in time.

Dry electrodes can be produced in even a small research laboratory, for instance by means of a 3D printer. Most of the commercial solutions combine dry, active, wearable and wireless electrodes in one system, thus giving rise to a fully operational mobile EEG system. However, some of the commercial products may be too expensive for small-size laboratories. Table 2 lists current commercial mobile EEG systems.

## 6. Conclusions

Currently, new advances in dry EEG electrodes give rise to unexpected fields of applications in addition to clinical applications. New aspects such as ease of usability and gel-free operation are first order priorities. Wet electrodes are considered the gold standard and further research must contrast dry and wet electrodes before claiming the adequacy of the former. The first difficulty is to reach agreement on the contrast methodology. Different dry electrode approaches are conceptually distinct and, in the literature, reports of performance have been carried out with non-homogenous methodologies, so their results cannot easily be discussed or compared. These studies and reports show evidences that additional work is needed before dry electrodes become an alternative to standard wet electrodes for the recording of EEG signals in clinical and other applications with long-term exposures

After reviewing most relevant dry-electrode approaches, we conclude that the evaluation of performance as well as the electromechanical characterization is a clear task that needs homogenization for a convenient comparison. In this regard, we propose a checklist of aspects that should be covered (see Table 3). As an example of application, Table 4 shows the most important details and characteristics of relevant studies.

## Figures and Tables

**Table 1.** Comparison of spike size, advantages and disadvantages of dry electrodes.

**Scale**	**Pros and Cons**	
nano	Similar impedance as wet electrodes No risk of infection Less artefacts due to motion	Invasive (piercing of SC) Non suitable for hairy sites

micro	Similar impedance as wet electrodes Less artefacts due to motion	Invasive (piercing of SC) Risk of infection Electrode fragility Non suitable for hairy sites

mili	No invasive No Risk of infection Suitable for hairy sites	Higher impedance than wet electrodes Artefacts due to motion

**Table 2.** Commercial EEG systems based on dry electrodes.

**Name**	**Purpose**	**Description**	**Vendor**
Sahara	BCI and General purpose	Dry, active electrode system that works for all frontal, central, occipital and parietal sites. Electrode composed of 8 pins made of gold alloy. Bandwidth: 0.1–40 Hz. When used with Nautilus: Sampling rate: 500 Hz. Up to 32 channels. 3-axis acceleration sensor.	g.tec medical engineering GmbH
Insight	BCI and General purpose	A 5 channel (plus 2 references) wireless headset to track and monitor brain activity and stream to mobile devices. Although the advertisement states it is a dry EEG system, the technical specifications state the sensors are made of semi-dry polymer. Bandwidth: 1–43 Hz, Sampling rate: 128 Hz, Wireless interface: Bluetooth 4.0 LE.	Emotiv
DSI 10/20	BCI and General purpose	Ultra-high impedance sensors (47 GΩ ). Up to 23 electrodes at a sampling rate of 960 Hz and a maximum bandwidth of 120 Hz. Suitable for locations with hair	Quasar
BrainBand XL, MindWave and others	BCI and for multimedia control	Dual sensor EEG unit (one active with adjustable positions). Bluetooth Connectivity. Sampling rate 512 Hz and bandwidth up to 50 Hz. Automatic with processing of attention, meditation and eye blink detection. Based in TGAM sensor by Neurosky. Not suitable for locations with hair.	MindPlay
XWave headset	BCI to control iPhone/iPad	Neuro Sky eSense Dry Sensor. Not suitable for locations with hair.	PLX Devices
Enobio	BCI and General purpose	UP to 20 channels at a sampling rate of 500 Hz. Wireless operation with Bluetooth and 50 nV of quantification step	Starlab
MindFlex	Electronic Game	Based on attention and meditation to control the vertical position of a plastic ball by activation of a fan underneath. It uses TGAM by Neurosky	Mattel
EEG headset	Wearable health monitor with EEG	8-channel EEG monitoring chipset. Each EEG channel consists of two active electrodes and a low-power analog signal processor with high input impedance (1.4 GΩ at 10 Hz)	Imec
ThinkGear AM (TGAM) EEG sensor	Brainwave sensor module for simper games	Non-contact dry sensor. Sampling rate 512 bits. Bandwidth 3–100 Hz. Operates at a minimum of 2.97 V. It works with Ag/AgCl, Stainless Steel, Gold, or/and Silver electrodes. It outputs attention, meditation and eyeblinks. Not suitable for locations with hair.	Neurosky
Dry Pad	BCI and General purpose	Reusable Ag/AgCl EEG pad electrode suitable for locations without hair. Electrode impedance 10–100 KΩ. The active version only needs a supply battery of 1.8 V. Small size (versions with 2–5 cm diameter circa.).	Cognionics
Flexible Dry EEG electrode	BCI and General purpose	Flexible and reusable (up to 30 sessions) Ag coated elastometer. Suitable for locations with hair. Electrode impedance 100–2000 KΩ.	Cognionics
Muse	Designed to manage stress with real time feedback.	Seven EEG electrodes built into a headband. Sampling rate 600 Hz.	Interaxon

**Table 3.** Description and evaluation of dry EEG electrodes.

Mechanical	Fundament (physical fundament of the approach, MEMS, non contact, *etc.*)
	Material (substrate, coating material)
	Dimensions (array, individua spikes)
	Fixation system (headband, helmet, *etc.*)

Electrical	Active (y/n) (need of on site active amplification)
	Impedance (response in frequency)
	Range of frequencies (for which the electrode is valid)
	Noise (noise figure)

Evaluation	Aplication (BCI, real-time outdoor use, clinical, *etc.*)
	Biosignals (EEG, EOG, EMG, ECG, *etc.*)
	Measure (*in vivo*, test signal, playback, *etc.*)
	Procedure/Paradigm (pysicophsyiological paradigm uses to elicit the features)
	Location (sites of the International 10-20 system)
	Features (ERP, Alfpha, μ-rhythm, SSVEP, *etc.*)
	Classification/Detection (e.g., in BCIs application)
	Comparison wet-dry (statistic method, e.g., correlation, MSE, *etc.*)

Usability	Dry (y/n)
	Cost
	Long-term (suitable for long-term monitoring?)
	Comfort (annoying, itching, need of tigh fixation?)
	Hairy sites (y/n)
	Others

**Table 4.** Details of relevant studies on dry electrodes and evaluation.

	**Mechanical**	**Electrical**	**Evaluation**
Griss 2002	**Fundament:** array of microneedles. **Material:** silver or silver-/silver chloride-coated microneedles glued onto a circular double-sided printed circuit board. **Dimensions:** micrometers. **Comfort**: Comfortable. Skin showed some redness.	**Active electrode:** No. **Impedance**: minimum 0.65–16 k at 1 kHz and 0.6 Hz. Unstable at frequencies below 0.8 Hz. **Noise:**	**Application:** anesthesia monitoring systems. **Biosignals:** EEG. **Measure:** *in-vivo*. **Procedure:** Comparison with Zipprep electrodes. **Features:** Spontaneous EEG. **Location:** forehead. **Evaluation: v**isual comparison dry Zipprep electrode.
Harland 2002	**Fundament:** Non-contact sensor. **Material:** metallic. **Dimensions:** disk of diameter 1 to 2 cm. **Comfort**: remote registering at 3 mm from the scalp, no physical contact.	**Active electrode**: Yes. I**mpedance**:10^15^ Ω and. **Noise:** 70 nV/Hz^−1/2^ at 1 Hz	**Application:** multichannel EEG monitoring and real-time imaging of the brain. **Biosignals:** EEG, ECG. **Measure:** *in vivo*. **Procedure:** Closed and open eyes. **Features:** Alpha and Beta. **Location:** P3 and O1: **Evaluation:** Visual plots of Alpha and Beta modulation. No dry-wet contrast.
Sullivan 2007	**Fundament:** Non-contact. **Material:** Metallic plate on the bottom of a PCB. **Dimensions:** A quarter dollar. **Comfort**: No physical contact, 3 mm gap. ECG records through clothing.	**Active electrode**: Yes. **Electrode impedanc**e: Not reported. **Noise:** 2 μVrms at 0.2 mm sensor distance, and 17 μVrms at 3.2 mm distance over 1–100 Hz frequency range	**Application:** Biopotential recording systems and human machine interfaces. **Biosignals:** EEG, ECG. **Measure:** *in vivo*. **Procedure:** Open-closed eyes paradigm**. Features:** Alpha. **Location:** back of the head, reference behind the ear. **Evaluation:** visual plots. No dry-wet contrast.
Oehler 2008	**Fundament**: Capacitive electrodes. **Material**: metallic electrode plate. **Dimensions**: Diameter of the electrode plate is 26 mm, sensor height is 15 mm. **Comfort**: Electrodes fixed by means of a helmet.	**Active electrode**: Yes. **Impedance:** 10^6^ GΩ. **Noise:** 2 μV/Hz^−1/2^ at 10 Hz and 70 nV/Hz^−1/2^ at 1 kHz.	**Application:** BCIs. **Biosignals:** EEG. **Measure:** *in vivo*. **Procedure:** Gazing at visual structured stimuli (checkerboards). **Features:** SSVEP. **Location:**O1, O2 and Oz, reference at FCz. **Evaluation:** Accuracy of SSVEP detection, ITR (bpm). No dry-wet contrast.
Matthews 2008	**Fundament:** A bioelectrode composed of 32 “fingers” capable of measurements through hair. **Material:** Metallic contact. **Dimensions:** US 5 cents approximately. **Comfort**: Use of harness. It permits subject motion.	**Active electrode:** Yes. **Impedance:** 10 MΩ per “finger”. **Noise:** 400 nV/Hz^−1/2^ (DAQ).	**Application:** Real time classification of workload during motion, Military applications. **Biosignals:** EEG, ECG, EMG, EOG. **Measure:** *in vivo*. **Procedure:** closing eyes. Classification of workload and engagement. **Features:** Alpha. **Location:** Cz, C3, C4, Fz, F3, F4, and Pz, reference at P4. **Evaluation:** Correlation with wet electrodes. Classification accuracy of cognitive workload.
Ruffini 2008	**Fundament:** Multiwalled carbon nanotube arrays. **Material:** Carbon without coating. **Dimensions:** Diameter of *ca.* 50 nm, length of 10–15 μm. **Comfort**: No side effects or pain even after 6 months.	**Active electrode:** Yes. **Impedance:** Not reported. **Noise:** It is low and rather similar to that of the commercial electrodes.	**Application:** General EEG recordings. **Biosignals:** EEG, ECG, EOG. **Measure:** *In vivo*. **Procedure:** Open-closed eyes paradigm. Auditory Evoked responses. **Features:** Alpha, N1 ERP. **Location:** Fp2, reference at nose. **Evaluation:** visual comparison dry-wet. Contrast hypothesis.
Dias 2010	**Fundament:** Array of 4 × 4 microtips. **Material:** silicon substrate coated with IrO. **Dimensions:** Each tip has a width in the range 150–200 μm, height of 100–200 μm, inter-microtip spacing of 2 mm. **Comfort**: Not reported	**Active electrode:** No. **Conductivity:** Similar to wet electrodes above 3 Hz. **Noise:**	**Application:** Human–machine interfaces. Suitable for electro-tactile stimulation. **Biosignals:** EEG, ECG, EOG, EMG. **Measure:** *In vivo*. **Procedure:** Eye movements, EOG measure. **Features:** EOG amplitude. **Location:** At the canthi of the eyes. **Evaluation:** Visual comparison dry-wet
Grozea 2011	**Fundament:** Flexible conductive bristles. **Material:** flexible metal-coated polymer bristles. **Dimensions:** 12 × 12 mm, and 10 mm long. **Comfort:** Pressure of the electrodes and the mounting frame found annoying. prickling sensation	**Active electrode:** No. **Impedance:** 80 KΩ (150–200 KΩ after 10 months of use). **Noise:**	**Application:** BCI. **Biosignals:** EEG. **Measure:** *In vivo*. **Procedure:** Eyes open/eyes closed conditions. Auditory evoked potentials. Odd ball paradigm and motor imagery. **Features:** Alpha rhythm. N100 AEP. P300, μ-rhythm (8–14 Hz). **Location:** P3, {Oz, Alpha}, {Fz, N100}, {Fz, Cz, P1, P300}, {Fz, C4, FC2, CP2, P3, μ-rhythm}. **Evaluation:** Spectral coherence (7–44 Hz) between dry-wet. T-test for detection of potentials.
Liao 2011	**Fundament:** Electrode with 17 spring contact probes. **Material:** BeCu. The probe head was coated with gold. **Dimensions:** 13 mm diameter, 20 mm height. **Comfort**: Not reported.	**Active electrode:** No. **Impedance:** Similar and better to wet electrodes on forehead and hairy site respectively. After 1 h, dry electrode impedance was better. **Noise:**	**Application:** BCIs, monitoring of human EEG states. **Biosignals:** EEG, EOG. **Measure:** Pre-recorded EEG data and *in vivo*. **Procedure:** Eye blinking. **Features:** Amplitude of EOG. L**ocation:** On the forehead (F10) and a hairy site (POz). **Evaluation:** Correlation with wet recordings.
Chin-Teng 2011	**Fundament:** Foam-based electrode. **Material:** foam covered by a conductive fabric and coated with Ni/Cu. **Dimensions:** 14 × 8 × 8 mm. **Comfort:** The foam electrode is soft enough to contact the skin properly.	**Active electrode**: No. **Impedance:** Similar and better to wet electrodes in forehead and hairy site respectively. After 1h dry electrodes impedance was better. **Noise:**	**Application:** monitoring of human EEG states, BCIs applications, clinical and research applications. **Biosignals:** EEG, EOG. **Measure:** Pre-recorded EEG data and *in vivo*. **Procedure:** EEG records and eye movements. **Features:** EEG and EOG amplitudes. **Location:** On the forehead (F10) and a hairy site (POz). **Evaluation:** Correlation with wet recordings.
Forvi 2012	**Foundation:** 8 × 8 pyramidal microneedles. **Material:** silicon. **Dimensions:** Contact surface 1 cm^2^.Micro scale. **Comfort**: Minimal invasiveness, easy and fast to use.	**Active electrode:** No. **Impedance:** 13 KΩ when SC is penetrated. **Noise:**	**Application:** monitoring of ECG signal in dynamic conditions. **Biosignals**: EEG, ECG, EMG. **Measure:** *In vivo*. **Procedure:** blinking eyes and in teeth grinding. **Features:** EEG, EMG, ECG. **Location:** EEG Fp1, Fp2, T3 and T4, ground at Fpz, reference at right mastoid. **Evaluation:** Visual comparison wet-dry.
Salvo 2012	**Fundament:** 3D printed dry electrode. **Material:** insulating acrylic based photopolymer. Coated with titanium and gold. **Dimensions:** millimeters. **Comfort**: Better than wet electrodes. It does not penetrate the SC.	**Active electrode:** No. **Impedance:** 1–3 KΩ and 2–5 KΩ at Fp1 and Fp2, respectively, whereas 4–7 KΩ and 1–3 KΩ are found at O1 and O2. **Noise:**	**Application:** Low cost biosignal applications. **Biosignals:** EEG, ECG. **Measure:** *In vivo*. **Procedure:** closed eyes. **Features:** Alpha. **Location:** O2, O1, Fp2, Fp1, reference at T6. **Evaluation:** visual comparison of dry *vs.* bridge electrodes.
Mota 2013	**Fundament:** Electrode with a gel reservoir inside. **Material:** Polymer coated with AgCl. **Dimensions:** 8 mm diameter, 11 mm height. **Comfort**: Not reported.	**Active electrode:** No. **Impedance:** 39 Ωcm^2^ at 10 Hz. 45 Ωcm^2^ at 1 Hz. **Noise:**	**Application:** Biopotential monitoring. **Biosignals:** EEG, EMG, ECG). **Measure:** \. **Procedure and Features:** Resting state EEG, Alpha activity EEG, Eye open/close test, Eye blink test. **Location:** Fp1, Fp2, O1, O2, ground at Fpz. **Evaluation:** RMSD, correlation, visual.
